# Community Health Agents and child health care: implications for continuing education

**DOI:** 10.1590/1980-220X-REEUSP-2021-0544

**Published:** 2022-04-11

**Authors:** Caroline Lopes Vieira, Valentina Barbosa da Silva, Elen Petean Parmejiani, Daniela Ferreira Borba Cavalcante, Maria Helena do Nascimento Souza, Marluci Andrade Conceição Stipp

**Affiliations:** 1 Universidade Federal de Rondônia, Porto Velho, RO, Brazil.; 2 Universidade Federal do Rio de Janeiro, Rio de Janeiro, RJ, Brazil.

**Keywords:** Community Health Workers, Education, Continuing, Child health, Agentes Comunitarios de Salud, Educación Continua, Salud del Niño, Agentes comunitários de saúde, Educação permanente, Saúde da Criança

## Abstract

**Objective:**

To understand the main situations faced by community health agents in relation to children’s health in the light of permanent education actions.

**Method:**

This is a research of qualitative approach, which used the Arc of Maguerez. Ten community health agents from a Primary Health Care Unit participated in the study. The following steps were addressed: observation of reality; identification of key points, and theorization. The speeches were recorded, transcribed, and their textual content was processed in the IRAMUTEQ software, using the Descending Hierarchical Classification.

**Results:**

Five classes were formed, which composed three thematic blocks named as follows: child’s social vulnerability in the territory; handling the child’s health record, and vaccination schedule.

**Conclusion:**

Unveiling situations that influence the work of community health agents is essential for continuing education, as this favors assumptions applicable to daily work with resoluteness in child health.

## RESUMO

**Objetivo:**

compreender as principais situações enfrentadas pelos agentes comunitários de saúde em relação à saúde da criança sob a ótica das ações de educação permanente.

**Método:**

trata-se de uma pesquisa com abordagem qualitativa, que utilizou o Arco de Maguerez. Participaram do estudo 10 agentes comunitários de saúde de uma Unidade de Atenção Primária à Saúde. Foram abordadas as etapas: observação da realidade; identificação dos pontos-chave e teorização. Os discursos foram gravados, transcritos e seu conteúdo textual foi processado no software IRAMUTEQ, utilizando-se a Classificação Hierárquica Descendente.

**Resultados:**

Formaram-se cinco classes, que compuseram três blocos temáticos nomeados da seguinte forma: vulnerabilidade social da criança no território; o manuseio da caderneta de saúde da criança e o calendário vacinal.

**Conclusão:**

O desvelar das situações que impactam no trabalho dos agentes comunitários de saúde são imprescindíveis para a educação permanente, por favorecer pressupostos aplicáveis no cotidiano do trabalho com resolutividade na saúde da criança.

## RESUMEN

**Objetivo:**

Comprender las principales situaciones vividas por los agentes de salud comunitaria en lo que se refiere a la salud del niño bajo la óptica de las acciones de educación permanente.

**Método:**

Es una investigación con abordaje cualitativo que utilizó el Arco de Maguerez. Participaron del estudio 10 agentes de salud comunitaria de un Centro de Atención Primaria a la Salud. Se abordaron las etapas: observación de la realidad, identificación de las cuestiones principales y teorización. Los discursos fueron grabados, transcritos y su contenido textual fue procesado en el software IRAMUTEQ utilizando la Clasificación Descendente Jerárquica.

**Resultados:**

Resultaron cinco clases que compusieron tres grupos temáticos nombrados de la siguiente manera: vulnerabilidad social del niño en el territorio, el manejo del carné de vacunación del niño y el calendario de vacunas.

**Conclusión:**

La revelación de las situaciones que impactan el trabajo de los agentes de salud es imprescindible para la educación permanente, una vez que puede favorecer premisas aplicables en el cotidiano del trabajo con resoluciones en la salud del niño.

## INTRODUCTION

In Brazil, the preferential contact of users with the health system takes place through Primary Health Care, which shall consider the individual in their singularity, complexity, in an integral way with the proper sociocultural insertion, always seeking to promote health of the individual and the community, with the prevention and treatment of diseases and the reduction of damage or suffering that may compromise their possibilities to live according to their potential and needs^(1)^.

Among the care and attention aimed at the community in Primary Health Care (PHC), the work of community health agents (*ACS*), who are professionals with the potential to support universal access to health for more vulnerable populations and communities who depend on publicly funded services^(2)^, stands out. International studies also emphasize the importance of *ACSs* as they are a link between the community and the health unit, which allows care longitudinality and the fight against conditions such as the current pandemic situation caused by the transmission of the new Coronavirus^(3,4)^.

In our experiences of professional practice, we noticed a certain lack of knowledge about their own challenges and the experiences *ACSs* face on a day-to-day basis regarding child health care. The issue this study elucidates refers to training and other qualifications, sometimes decontextualized of the main situations these professionals face, proving to be inadequate to the contexts they experience, as well as occasionally neglected, which precludes the achievement of the real needs of continuing health education (*EPS*) in their daily practices.

This problem is not specific of a Brazilian region. In fact, it covers large part of the national and international territory, intertwined with a reality of low investments and no public policies aimed at building an EPS based on methodologies that value the workers’ previous knowledge and can ensure quality in health care for the community^(5)^.

A study carried out in the United Kingdom corroborates this observation, highlighting that the implementation of an ACS training program, even if of an emergency nature, was a potential model of effective support for the health of families in the long term^(4)^. Another study investigating the current understanding of ACS programs in countries with different economic backgrounds, such as Greece, Mexico, the United States of America, including Brazil, pointed out that the adequate amount and type of training required by ACSs shall be related to the context of the local health system, with its pre-existing knowledge and the skills expected from these workers^(6)^.

Educational practices shall be consolidated at local levels, especially with regard to the analysis of lived experiences, valuing and strengthening collaborative practices. The work of the ACSs requires interprofessional skills, given the integrality of users and the community, who should have their needs met by the Family Health teams (FHt), since such strategy was designed to bring users closer through interprofessional work^(7)^.

Thus, for the ACSs to carry out their duties effectively, qualification is required concerning the identification, guidance, referral, and monitoring of users, aiming at solving the health demands of their territory. Providing a qualification environment for agents is crucial to achieving maximum efficiency. Therefore, several inputs have to be interconnected, so that the ACSs become more productive in their roles^(8)^.

Considering the relevance of the actions developed by the ACSs in child health care, the following question arises: which situations problematized by the ACSs are considered in the construction of EPS actions in child health? Thus, the objective of this study was to understand the main situations faced by community health agents in relation to children’s health in the light of permanent education actions.

## METHOD

### Design of Study

The qualitative research approach was used for the understanding of situations of ACSs’ daily work in child health care, as well as the understanding of critical aspects, beliefs and values related to this context^(9)^. We implemented the problematization methodology with the Arc of Maguerez, through discussion and problematization as a guiding way to investigate ACSs daily work, consisting of five steps: observation of reality; identification of key points; theorization; solution hypotheses and application to reality^(10)^. In the present study, the steps “observation of reality”, “identification of key points” and “theorization” were used.

### Local

The study location was a Family Health Unit in the city of Porto Velho, Rondônia, inserted in the context of the municipality’s PHC, being one of the 20 basic units serving the urban area, located in the East Zone of the city. This unit was chosen because it is a place where reports had already been observed among the ACS regarding the difficulties related to child health care and the need for educational interventions for the workers. Moreover, the aforementioned unit constitutes an important scenario for the integration with teaching, through an agreement for the insertion of students from the undergraduate course and residency programs at the Universidade Federal de Rondônia, improving the practices of both professionals and students.

### Population

Ten ACSs, of both sexes, who work in two of the four FHt of a Family Health Unit, located in the East Zone of the city of Porto Velho, Rondônia, participated in this study. The selection criteria were: ACSs working at the FHt for a period of one year or more and who were in full exercise of their care functions. For exclusion criteria, we considered the ACSs on leave, vacation, and those who did not develop care activities during the period studied.

### Data Collection

Data collection for this study took place in the first stage of the Arc of Maguerez, that is, the observation of reality, covering the period from August to November 2020. Participants were initially approached through individual and in-person contact with the principal researcher, who is a nurse and, at the time of collection, was working as a student of the second year of the multiprofessional residency in family health at the Universidade Federal de Rondônia. This contact was possible because the researcher had been performing her duties with the FHS team for a year, which allowed for the dissemination of the research, its objectives, and other necessary information for the reception and approximation with the participants. Subsequently, contact was maintained via email and telephone.

After this moment, we created a virtual workgroup through the WhatsApp application^(11)^, as well as an in-person working group, which took place on the premises of the Family Health Unit, according to the ACSs’ work agendas. A total of 25 participants were added to both groups, covering all those working in that scenario. Then, the invitation was made and due clarification about the study was given, so that 10 agreed to participate; nine were away and six refused to participate. The main reason reported for non-adherence to the study was the incompatibility of the work schedule, even with several attempts to reconcile compatible schedules. The online workgroup had a double intention: while bringing the workers closer, it also provided a precise environment for the collection of information about the ACSs’ daily routine, through the speech of the deponents. The researcher was the one who led and facilitated all the dynamics during data collection, ensuring a space for permanent education for the safe conduction of the groups.

A constant two-step semi-structured guide was attached to the online workgroup. The first step was related to ACS characterization, consisting of closed questions about variables – age, sex, education, work and qualifications. The second stage had three open questions, which raised the following issues: What are your experiences related to child health? What situations in daily work have caused difficulties in the practice of child health care? How have you carried out the activities in the face of the difficulties encountered? The first stage of the guide was answered in the online working group itself and the open questions were discussed and dialogued among the participants through the audio recording and writing resources in the WhatsApp application, and these moments of discussions were extended during the meetings of the in-person working group.

A pilot test of the interview guide was not carried out, since the production of data generated in the work groups facilitated the approximation and understanding of the participants about the triggering questions, which mobilized sufficient discussions to reach the proposed objective. Thus, there was one meeting with the online workgroup and three meetings with the in-person working group, with an average duration of 60 to 120 minutes. All meetings were recorded through digital recording and later fully transcribed. The pandemic made ACSs in-person participation difficult, due to absences, because they were part of the risk group, and due to the need to reduce in-person moments.

### Data Analysis and Treatment

For data processing, we used the second stage of Arc of Maguerez, the identification of key points, as a reference. Thus, we prepared the transcribed material, as well as the coding, transforming it into a text *corpus* to be processed in the IRAMUTEQ software®-Interface of *R Pourles Analyzes Multidimensionnelles de Texteset de Questionnaires*. This software performs the analysis of lexical roots and offers the contexts in which the classes are inserted, according to the text segments (TS)^(12)^. Then, we proceeded with the stage of class analysis, including the stages of description of the themes expressed in the narratives, which allowed the definition of categories and the extraction of meanings from the data, presented with support from the literature. The study was conducted in accordance with the consolidated criteria for qualitative research reports – COREQ^(13)^.

### Ethical Aspects

The study complied with the ethical and legal aspects of the research in compliance with Resolutions 466/2012 and 580/2018 of the National Health Council, being approved by the Research Ethics Committee of the Universidade Federal de Rondônia, under opinion number 3.720.871/2019. To ensure anonymity, the text segments presented in the results were identified as Part 1, Part 2, Part 3… Part 10, not following the order of the testimonies in the virtual and in-person working groups.

## RESULTS

Among the 10 study participants, eight were women, in the predominant age group between 34 and 59 years old. It should be noted that the excerpts referring to six participants are included in this manuscript, but these statements were inserted as they contemplate the speeches of all the participants in the classes analysis, as well as in the construction of the thematic blocks. Regarding the professional profile and training, five have higher education (nursing, pedagogy, physical education, information technology, and one did not inform his/her area); of these, two have a graduate certificate or are studying for it; three reported having technical training and one reported having a high-school level. With regard to technical training, two participants informed that they were nursing technicians and the other participant did not specify his/her technical area.

Regarding the length of service as ACS, four workers have up to 11 years and six have more than 15 years of work. Regarding qualifications, after entering the job, five reported having taken the introductory course for ACS. Regarding child’s health, seven of them reported having participated in educational activities aimed at ACS qualification, but only one participated less than five years before; among these, the topics mentioned were: role of the ACS, territorialization, child growth and development, and vaccination. In the analysis of the reports and discussions in the groups, online and in-person work, we got a text *corpus* consisting of 10 texts which, after IRAMUTEQ processing, through CHD, resulted in 219 text segments, distributed in five classes, having used 169 text segments (77.17%), which represents good quality of the processed material^(12)^.

**Figure 1:**
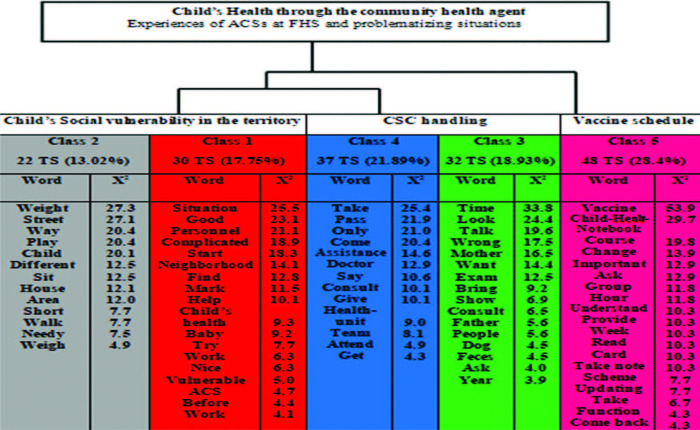
Dendrogram referring to the distribution of the word of the classes according to the Descending Hierarchical Classification, adapted by the authors from the report provided by the *software* IRAMUTEQ – Porto Velho, RO, Brazil, 2021.

Source: Prepared by the authors based on the Iramuteq report.

Figure 1 shows the composition of the classes based on the enunciation of words, according to their qualitative (semantic) and quantitative (p < 0.05) relevance, being the basis for data analysis and interpretation according to their application in the context of the classes. The classes were grouped into thematic blocks, according to the partition of the text *corpus and* the due approximation between classes, which generated three thematic blocks, presented below.

### First Thematic Block: Social Vulnerability of the Child in the Territory

This block consists of classes 1 and 2, which totals 30.3% of the text segments and reveals the experiences of the participants in the social context of the children followed. In class 1, we observe the elements that point to the socioeconomic vulnerability of the population, which culminates in difficulties in accessing health services. The main difficulties mentioned by the participants were related to geographic barriers, the process of scheduling appointments and the lack of financial resources to travel to the unit. However, ACSs state in their reports that even in the face of these obstacles, they continue to provide guidance on the appointments and services offered by the FHt to those responsible for the children.

*We had a patient who was disabled and she had a small child and had a baby. For her to come from there to here, the health basic unit, it was very complicated, her husband worked as a part-timer and had fallen off the roof and had a broken leg* (Part 1).

*And you arrive at the residence and see that precarious situation, the people trying to survive (Part. 2).*

*We will see several degrading situations, without health in terms of hygiene, sewage, this will be bad for a child to be playing nearby (Part. 04).*

In class 2, the participants expose their experiences and difficulties in the territory where they carry out child growth and development monitoring, through the measurement of weight and height. A difficulty related to weight measurement is the absence of an appropriate scale for the child’s age, which impairs the proper recording in the Children’s Health Handbook (CSC) chart.

*My experience was only with the monitoring and development of the child, which was weighing and checking the child’s height; to know her vaccination schedule was delayed, if it wasn’t, if she was underweight, malnourished or overweight. And also in 2004, as soon as we entered, we made the multimixture, which was prepared for these low-weight children* (Part 5).

Therefore, the thematic content of this block reveals the absence of social and environmental conditions favorable to the basic health condition of the territory, which, in turn, affect the work of ACSs.

### Second Thematic Block: Handling the Child Health Handbook – CSC

This block refers to the facilities and problems reported in the handling of the CSC, being formed by classes 3 and 4. In this block, the participants reveal the recognition of the role of the ACS as a facilitator of the child’s access to the health service, which is provided through the bond and the approximation of the community with the health actions promoted by the FHt, in which the Child Health Handbook is the instrument used for this purpose.

Class 3 evidenced the lack of ACS qualification for the management of the Child Health Handbook, as difficulties were pointed out in guiding the notes in the CSC regarding appointments, follow-up visits with nurses, doctors and nursing technicians, making ACS fearful of propagating wrong guidelines, especially about the vaccination schedule. As a result, daily work may be permeated by fear and insecurity in relation to child health care.

*Now for me to look there [in Child Health Handbook] and say; we don’t know, but we already knew, before there was training (Part. 02).*

*Regarding the graph of Child Health Handbook it’s all based in the eye, because the child has to be weighed to be classified as at the right weight or below (Part. 04).*

*There are things that patients say to us, I warn: ‘when you go to the doctor’s appointment, say this to him, not to me, I won’t be able to explain everything’*. (Part 3).

Class 4 showed the difficulties that the ACSs find when developing their work with other team professionals. Among them, they point out the difficulty in getting medical care for the needs of the child identified in the territory, sometimes due to the absence of the professional, the overcrowded schedule, and the low coverage of the FHt, generating an increase in spontaneous demand. In contrast, they emphasize the importance of consultations, home visits, and group activities, which are developed by the team.

*I prefer to take the team to the patient’s house, who live with a lot of people, and it’s not because a lot of people live in the house, but I’ve already noticed that when I’m alone I can’t say anything to the family* (Part 5).

*And we can’t be going to the residency to make a bond, see how they are, because I moved to another area, my doctor and my nurse will never accept or embrace this family, who is now in an uncovered area, I feel sorry and the family call me* (Part 5).

### Third Thematic Block: Vaccination Schedule

This block was consolidated by the grouping of words that formed class 5, consisting of testimonies related to preventive actions on the immunization of children in the context of the territory during the ACS home visit, referred to as a moment of listening, observation, and exchange of information. The text segments present in class 5 show difficulties experienced by the participants, who mention the qualification deficit regarding the child’s vaccination schedule. ACSs reported difficulties in identifying and guiding the population about the vaccination status of children in the territory based on the current annual schedule.

*One thing I can’t see is the Child Health Handbook because a lot has changed in the vaccine. If you ask me, ask me to look at the child’s Child Health Handbook, I don’t know how to (Pat. 03).*

*I open the Child Health Handbook and look, if the missing vaccine is recorded in pencil, I say it is late, but if there is nothing recorded, I advise going to the vaccine room and checking. Because there are some vaccines that have changed and I really don’t know (Part. 06).*

The ACSs report that they had previously had courses and training on vaccines and a mirror of the vaccination card was provided by the service management, which supported their work on this topic. According to the ACSs, this method facilitated the control of the children’s immunization schedule, in addition to ensuring greater attention to the care of the child and its vaccination.

*In the past, when it changed, I already had a nurse who said: “guys, the vaccination schedule has changed. The nurse gave a mirror card of the vaccines for each age group, so as much as I didn’t understand, it was written* there (Part 1).

Currently, ACSs seek help from the local vaccinator to assist them in identifying the vaccination status or searching for such information on the internet. However, due to constant changes in the vaccination schedule, these professionals experience difficulties and, thus, a deficit in the continuous EPS process is observed.

## DISCUSSION

The first action, after the constitution of the working groups, was the recognition of the participants who live and work in the territory. Half received an introductory course for ACS and the majority received qualification on child health more than five years before, mainly on the role of ACS, territorialization, child growth and development, and vaccination. Regarding these characterization variables, a study carried out in Kenya identified that age and educational status are likely to influence the performance of ACSs in carrying out their duties. In the findings of this study, opportunities for qualification and updating proved to be key factors for the role of ACS in child health care, and shall take place on a permanent basis, ensuring significant learning, awareness of demands, and the development of resolute actions^(14)^.

Social and environmental conditions also proved to be influential in the ACSs’ daily work. As they belong to the community, the ACS facilitates geographic access to the health service, through home visits. However, investments are required in transport infrastructure and in the expansion of health services, aiming at expanding coverage and facilitating access to PHC. Geographical barriers and access to health services are common challenges in several countries, especially in low-income countries^(15)^.

The issue of social vulnerability has several implications for children’s health, reflecting on the impairment of child development and their mortality rates. A study identified individual and contextual health care risk factors in determining infant mortality in 27 Brazilian capitals, pointing out that maternal socioeconomic vulnerability factors mediate the biological factors that determine infant mortality in Brazil^(16)^. In the territory, ACSs are faced with situations that require complex responses related to the fields of health and social and human sciences. To achieve resolution in the work of the ACS, support and interprofessional and intersectoral work are required, so that the problems requiring interventions that go beyond the governability of the health area are mitigated^(17)^.

The perception of these professionals about the problems related to social vulnerability is in line with the recognition of the health-disease process, according to the model on the Social Determinants of Health, correlating biological aspects, lifestyles and habits, social and community networks, living and work conditions, as well as the general socioeconomic, cultural and environmental conditions^(18)^. Therefore, given the complexity faced in the territory, as an integral subject of the community, and at the same time representative of the health service, ACSs identify the vulnerabilities associated with the social determinants of health, but find it difficult to face them^(19–20)^.

Faced with ACSs’ difficulties regarding the filling out and handling of the CSC, it is important that there are EPS actions to work on this theme. In child monitoring, the Child Health Handbook stands out as an important tool for controlling these actions in the community, being an essential health record at the monitoring of child up to 10 years of age. It contains information of child’s identification, health guidelines, and information related to children’s rights, as well as birth records, growth and development monitoring, vitamin supplementation, and vaccination schedule. Such records are the responsibility of health workers^(21–22)^. A study carried out in the city of Porto Velho, with 2,483 children aged up to five years, showed that only 25.5% of the CSC were satisfactorily fulfilled. The deficit in filling the Child Health Handbook reflects the fragility in monitoring integral growth and development, especially in early childhood^(23)^.

Another important issue is ensuring access to materials and work instruments, such as scales and tape measure, so that the ACSs develop their surveillance actions related to child growth. In Indonesia, a study compared ACSs’ skills before and after training for anthropometric measurements, highlighting the importance of monitoring growth through ACSs’ training and regular updating for a standardized and qualified anthropometric measurement^(24)^. In relation to measurement, it is also important that the ACS knows how to recognize the signs of danger in the child and the main parameters used, as stated in the Child Health Handbook^(22)^.

In this context, the EPS is an important tool for the qualification of ACSs. At the FHS, they have an important role in the teaching-learning process of the professional, setting up a space for problematizing the “making health”, providing opportunities for the re-signification of their experiences in the territory, strengthening the team’s work process and producing strategies for the transformation of the reality. However, it is necessary for the teams to be consistent and for the local permanent education center to be strengthened so that EPS actions can continue^(19)^.

To provide access to essential health promotion, prevention and recovery services, greater investments in PHC are needed, prioritizing the most vulnerable regions. Maternal and child health is included in the goals of global action on the Sustainable Development Goals, Agenda 2030, of the United Nations. Universal health coverage and the reduction of mortality from preventable causes of mothers and children make up some of the goals for achieving goal 3 – Health and well-being^(15)^.

Regarding the vaccination schedule, we inferred that for this study the instructions on vaccine are one of the most emerging actions in the practice of ACSs and this emphasizes the importance of this professional for vaccine uptake. However, the lack of knowledge about the current vaccination schedule and elementary information about vaccines has caused what is called Missed Vaccination Opportunities^(25)^, resulting in low vaccination coverage and greater susceptibility in the community.

The exchange of knowledge in the field of health, between professionals and users, must take place through EPS and health education. The ACSs have health education as the main axis of their work^(26)^. However, fear and insecurity in carrying out health education work indicate a lack of preparation and knowledge, related to the absence or fragility of the service’s EPS actions. It should be emphasized that for the effectiveness of health work, referring to EPS, and health work related to popular health education, the use of dialogic and participatory teaching-learning methodologies is essential, aiming at reaching an integral and equitable assistance to the individual and the community^(24)^.

Educational work on health requires critical reflection, aiming at the sharing of knowledge, the contribution for the population to recognize their situation of risk and the promotion of mobilization to guarantee social rights. In short, the interactivity between social subjects, aiming at social transformation in health^(27)^.

The qualification and EPS of the multidisciplinary team and the ACS are important means for redirecting health actions beyond curative practices. The ACS qualification actions need to make use of innovative teaching-learning methods that support the reflective process and have the student as the main actor, focusing on the development of skills and proactivity^(21)^. A study reaffirms the need to build public policies aimed at qualifying ACSs so that they have the competence to act in different contexts that build and express the health-disease process. For this, one of the paths indicated is the resumption of the technical training of the ACS, to be implemented in an integral way and offered to all^(28)^.

It is necessary to integrate the triad “teaching, service, and community” through educational institutions and their research programs, such as: undergraduate courses; the Education through Work Program (PET); university extension programs; medical and multi-professional residencies, among other teaching initiatives^(29)^. Always having a focus on health surveillance and EPS is essential for the creation of intervention with greater resoluteness on the care related to the health of the community, especially child’s health. The dissemination of this study can certainly contribute to the formulation of both local and national strategies, since the situations intersect with several other places of action in Primary Health Care.

## CONCLUSION

With the formation of the group, it was possible to understand the experiences, the difficulties, the facilities in the work of the ACSs, as well as the recognition of the team itself and the community that they assist. Regarding the situations faced by the ACS, we analyzed a set that revealed some problems that, on several occasions, hinder the work with child health care in the territory of the Primary Health Care. These situations mainly showed the formation of a bond with the community, in the desire to carry out adequate health guidelines and to update the vaccination schedule.

The EPS actions are fundamental to qualify ACSs to work effectively with the child’s health, overcoming the difficulties in the biopsychosocial plan. We here recognize the role of EPS as a protagonist of substantial changes in the context of the family health strategy, promoting the qualification of the FHt, so that its actions result in comprehensive care for the health of the child, being able to achieve more resolution, contributing to the formation and the possibility of transformation and resignification of this reality.

## ASSOCIATE EDITOR

Ivone Evangelista Cabral
